# Labile organic carbon pools and enzyme activities of *Pinus massoniana* plantation soil as affected by understory vegetation removal and thinning

**DOI:** 10.1038/s41598-017-18812-x

**Published:** 2018-01-12

**Authors:** Yafei Shen, Ruimei Cheng, Wenfa Xiao, Shao Yang, Yan Guo, Na Wang, Lixiong Zeng, Lei Lei, Xiaorong Wang

**Affiliations:** 1 Key Laboratory of Forest Ecology and Environment, State Forestry Administration, Research Institute of Forest Ecology, Environment and Protection, Chinese Academy of Forestry, Beijing, 100091 China; 2 Co-innovation Center for Sustainable Forestry in Southern China, Nanjing Forestry University, Nanjing, 210037 China

## Abstract

The effects of forest management on carbon (C) sequestration are poorly understood, particularly in the Three Gorges Reservoir area. We aimed to identify the effects of forest management on C sequestration in *Pinus massoniana* plantations. An intact control forest (CK), a site undergoing regular shrub cutting with the simultaneous removal of residues (SC), a site under low-intensity thinning (LIT), and a site under high-intensity thinning (HIT) were compared for soil labile organic carbon (LOC), related enzyme activities, and soil characteristics. Soil organic carbon (SOC) significantly decreased in the HIT treatment as compared with that in the CK treatment. Soil EOC, DOC, MBC contents in treated plots were higher than those in the CK treatment; particularly, the HIT treatment significantly increased those values in 0–10 cm layer. Thinning resulted in a decrease in cellulase and amylase activities, but an increase in invertase activity. In addition, the SOC content was significantly correlated with four enzymes activities and LOC components, which suggested that the soil LOC components and enzymes activities were sensitive to the changes of SOC. Our results suggest that high-intensity thinning treatment in *Pinus massoniana* plantation could significantly decrease the SOC content and lead to an increase of LOC components.

## Introduction

The forest ecosystem accounts for approximately 73% of the terrestrial soil carbon (C) pool, which is an important part of the global terrestrial ecosystem^1^. Thus, the forest ecosystem plays a crucial role in the C cycle^2^. A major focus of forest management is to promote the increment of C pool^3–5^. In soil, according to their mean residence times, soil organic carbon (SOC) can be divided into recalcitrant and labile components. Management practices have little effect on recalcitrant components because of their longer turnover time in soil^6^, while soil labile organic carbon (LOC) fractions are more responsive to changes in forest management strategies. Although soil LOC fractions make up a relatively small part of SOC^7–9^, they can serve as indicators of minor changes in SOC.

Responses of soil LOC components are often indicated by easily oxidised organic carbon (EOC), dissolved organic carbon (DOC), and microbial biomass carbon (MBC)^6,10^. Previous studies have shown that EOC, DOC, and MBC contents affect C sequestration capacity of soil and the emission of greenhouse gases^11^ thus indicating that they are important sources of C that are released from the soil to the atmosphere and aid in the decomposition of recalcitrant C^12^. Thus, soil LOC fractions in forests are imported for maintaining balance in the soil C pool under different forest management strategies. Moreover, the activities of enzymes related to the soil C cycle (e.g., invertase, amylase, and cellulase) participate in the SOC decomposition and indicate the status of the available C resources. Therefore, these enzyme activities can contribute to our understanding of the variations in SOC in response to forest management^13,14^.

Thinning is a common strategy of forest management used in plantations forests, and it is a tool for controlling the species composition^15^. Thinning commonly decreases SOC by reducing litter inputs into soil and possibly accelerating decomposition rates owing to change in microclimate^3,12^ and their change degree of SOC among different soil layers were varied^12^. Understory vegetation can modulate SOC by affecting the soil characteristics and changing the organic inputs and the leaching of dissolved organic matter^16^. Different forest management strategies can change soil temperature and moisture, and the composition of aboveground vegetation, thereby influencing C and nutrient cycles in forests^17–19^. For example, whole-tree harvesting has the most significant effect on the soil C pool, because it causes direct damage to vegetation via SOC release, increased soil temperature, and accelerated erosion^20,21^. Accumulating evidence shows that for most tree species, the effect of forest thinning on SOC dynamics is complex, and that the intensity and method of forest management affect the degree to which microclimate and residual vegetation composition are affected, thereby affecting SOC sequestration^3,22^. However, studies on forest C storage under different forest management strategies have produced contradictory conclusions^23,24^. It has been reported that decreasing tree density in stands decreases total forest C stores^25,26^, whereas others have found that this action can maintain or increase live tree C due to the increased growth rate of trees grown at lower densities^27^, especially in the case of long-term responses to thinning^28^. Short-term studies have revealed that thinning consistently decreases aboveground C^26,29^, indicating that low densities of small trees do not fully offset the loss of C^30^. Thus, it is still unclear whether forest management strategies are compatible with the purpose of increasing forest C storage for climate change mitigation. In addition, exploring the effects and mechanisms affecting forest management strategies on SOC sequestration is a key research area in both forestry and C cycling science, while relatively less attention was paid to the effect of short-term forest management activities on soil LOC fractions^31^.

Plantations are a key component of global forest resources, and play an important role in sustainable forest management^32–34^. In China, pure plantations forests constitute approximately 80% of all plantations^16^. *Pinus massoniana* is the main tree species used for afforestation in South China. It plays a major role in providing forest resources and ecological services^35^, and it covers the largest area (67.83 × 10^4^ ha) in the Three Gorges Reservoir area located in subtropical China^36^. However, due to anthropogenic effects and the complex terrain in this region, the soil C content is relatively low^37^. Thus, in recent years, recreating forest structures and optimizing the use of soil by forest management activities were a major focus of the sustainable management of *Pinus massoniana* aimed at maintaining ecosystem sustainability and soil productivity^12^.

In this study, the objective was to assess the effect of forest management in term of SOC, LOC fractions, and activities of related enzyme at three mineral soil layers (0–10 cm, 10–20 cm, 20–30 cm). The aim of the project was to investigate (1) how different forest management treatments (0%, 15% and 70% stem thinning and understory vegetation removal) influence the contents of SOC, soil LOC fractions (i.e. DOC, MBC, and EOC), and related enzyme activities (i.e. cellulase, amylase, invertase, and catalase) and (2) whether relationships exist among soil LOC fractions, enzyme activities and other soil characteristics (soil pH and fertility characteristics). Our hypotheses are that 1) thinning and understory vegetation removal treatment will decrease the SOC, enzyme activities and increase LOC pools, and 2) the soil LOC fractions are linked to enzyme activities and partial soil characteristics.

## Results

### Soil chemical properties

Soil total nitrogen (TN), total phosphorous (TP), available potassium (AK), NH_4_^+^–N, and NO_3_^−^–N contents decreased with increasing soil depth while soil pH increased with increasing depth, although significant difference were only observed in the soil TN content and pH among the soil layers (*p* < 0.05). Besides this, in the 0–10 cm soil layer, TN, total potassium (TK), AK and NO_3_^−^–N contents were significantly lower in the three treated plots than those in the control plots (*p* < 0.05). In the 10–20 cm soil layer, AK and NO_3_^−^–N contents in the three treated plots were significantly lower than those in the control plots (*p* < 0.05). In addition, TN content in the SC treatment plots was greater than those in the thinning (LIT, HIT) plots in 0–10 cm and 10–20 cm soil layer (*p* < 0.05). In the 20–30 cm soil layer, NH_4_^+^–N and NO_3_^−^–N contents in three treated plots were significantly lower than that in the control plots (*p* < 0.05) (Table 1).

**Table 1:** Soil chemical properties at three soil depths in the four forest management treatments (mean value ± standard error; n = 3). Significant differences among different soil layers subjected to the same treatments are identified with A, B, and C (*p* < 0.05). Significant differences among different treatments of the same soil layer are identified with a, b, c, and d (*p* < 0.05), based on the analysis of variance.

Treat- ments	Soil depth (cm)	Soil pH	TN	TP	TK	AP	AK	NO_3_^−^–N	NH_4_^+^–N
CK	0–10	5.85 ± 0.02 Aa	1.65 ± 0.01 Aa	0.21 ± 0.01 Aa	17.05 ± 0.12 Aa	0.84 ± 0.10 a	184.68 ± 2.19 Aa	17.79 ± 0.89 Aa	43.00 ± 3.51 Aa
	10–20	5.92 ± 0.06 Ba	1.15 ± 0.01 Ba	0.18 ± 0.01 Ba	16.76 ± 0.13 Ba	0.99 ± 0.17 a	145.83 ± 2.80 Ba	11.26 ± 0.99 Ba	37.34 ± 3.48 Ba
	20–30	6.07 ± 0.05 Ca	0.97 ± 0.01 Ca	0.17 ± 0.01 Ba	17.13 ± 0.10 Ba	0.98 ± 0.17 a	136.58 ± 0.47 Ca	5.40 ± 0.0.37 Ca	24.66 ± 0.56 Ca
SC	0–10	6.02 ± 0.05 Aa	1.57 ± 0.02 Ab	0.20 ± 0.01 Aa	18.21 ± 0.15 Ab	1.23 ± 0.17Aa	146.45 ± 2.04 Ab	11.65 ± 0.22 Ab	45.66 ± 0.69 Aa
	10–20	6.16 ± 0.03 Ba	1.16 ± 0.01 Ba	0.19 ± 0.01 Ba	18.08 ± 0.25 Bb	2.35 ± 0.29 Bb	126.18 ± 1.28 Bb	4.48 ± 0.14 Bb	18.96 ± 0.23 Bb
	20–30	6.33 ± 0.03 Ca	0.90 ± 0.01 Cb	0.18 ± 0.01 Ca	19.52 ± 0.19 Ca	1.57 ± 0.4 ABab	110.43 ± 2.47 Cb	2.75 ± 0.34 Cb	12.76 ± 1.28 Cb
LIT	0–10	6.17 ± 0.02 Aa	1.37 ± 0.04 Ac	0.19 ± 0.01 Aa	16.54 ± 0.21 Ac	2.11 ± 0.39 b	140.60 ± 3.29 Acb	9.73 ± 0.42 A c	50.69 ± 2.91 Ab
	10–20	6.25 ± 0.03 Ba	1.04 ± 0.02 Bb	0.17 ± 0.01 Ba	17.14 ± 0.08 Ba	1.46 ± 0.92 ac	127.40 ± 2.50 Bb	2.57 ± 0.10 Bc	14.79 ± 0.47 Bc
	20–30	6.48 ± 0.02 Ca	0.85 ± 0.0 1 Cc	0.17 ± 0.01 Ba	17.70 ± 0.24 Aa	2.51 ± 0.61 b	126.25 ± 3.29 Aa	2.45 ± 0.13 Bb	10.99 ± 0.28 Cb
HIT	0–10	5.97 ± 0.05 Aa	1.49 ± 0.01 Ad	0.19 ± 0.01 Aa	16.43 ± 0.16 Acd	0.82 ± 0.70 Aa	130.78 ± 0.52 Ac	5.35 ± 0.19 Ad	35.79 ± 3.29 Ac
	10–20	6.07 ± 0.03 Ba	1.09 ± 0.02 Bc	0.17 ± 0.01 Ba	15.83 ± 0.34 Bc	1.09 ± 0.10 ABad	82.90 ± 1.60 Bc	4.90 ± 0.58 ABb	34.51 ± 2.41 Aa
	20–30	6.20 ± 0.04 Ca	0.96 ± 0.01 Ca	0.18 ± 0.01 Ca	16.58 ± 0.05 Ca	1.91 ± 0.90 Bab	71.68 ± 1.57 Cc	3.50 ± 1.56 Bb	29.25 ± 1.50 Bc

### LOC fraction

**Figure 1:**
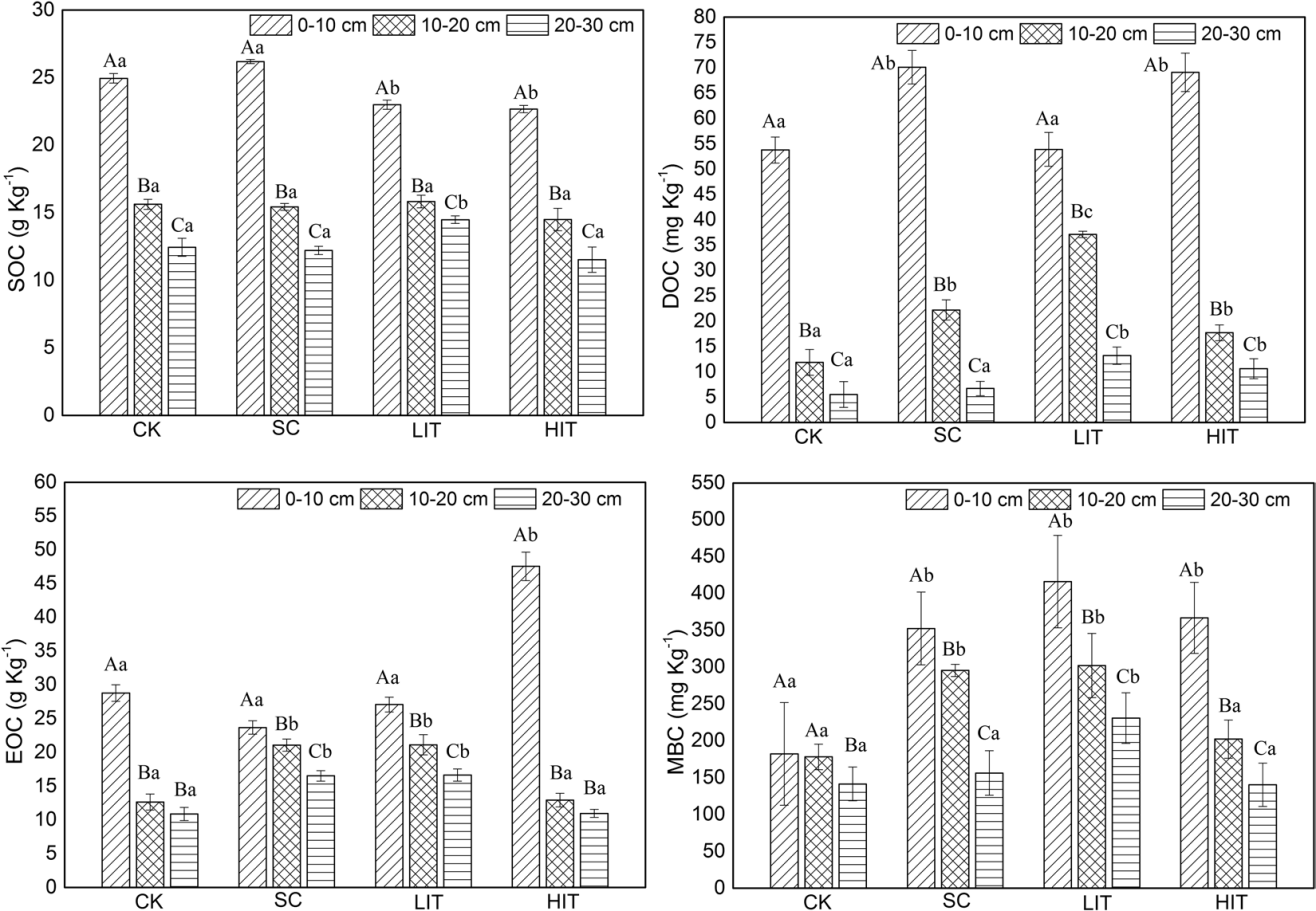
Soil LOC fractions in the four forest management treatments. The three columns in each treatment represent the quantities in soil LOC content at different soil depths. Significant differences among different soil layers subjected to the same treatments are identified with A, B, and C (*p* < 0.05). Significant differences among different treatments of the same soil layer are identified with a, b, c, and d (*p* < 0.05), based on the analysis of variance. Values are means ± standard error (n = 3).

SOC, EOC, DOC, and MBC decreased with increasing soil depth, and significant difference were only observed in the SOC and DOC contents among the three soil layers (*p* < 0.05). The EOC content in the 0–10 cm layer was significantly higher than that in the other soil layers (*p* < 0.05). In the 0–10 cm soil layer, SOC content under the LIT and HIT treatment were significantly lower than those in the CK and SC plots, but in 0–30 cm soil layer, significant difference between the HIT and CK treatment was only observed (*p* < 0.05). The contents of DOC under the SC and HIT treatment were significantly higher than those in CK and LIT treatment in 0–10 cm soil layer (*p* < 0.05), and the corresponding values in the 10–20 cm layer were significantly higher than those in the CK (*p* < 0.05). The content of EOC in the 0–10 cm layer was significantly higher in the HIT treatment than that in other three treated plots (*p* < 0.05). In addition, the content of MBC in the three treated plots were higher than those in the CK, and, in particular, there was only a significant difference between LIT and CK treatment observed in three soil layers (*p* < 0.05) (Fig. 1).

### Soil enzyme activity

**Figure 2:**
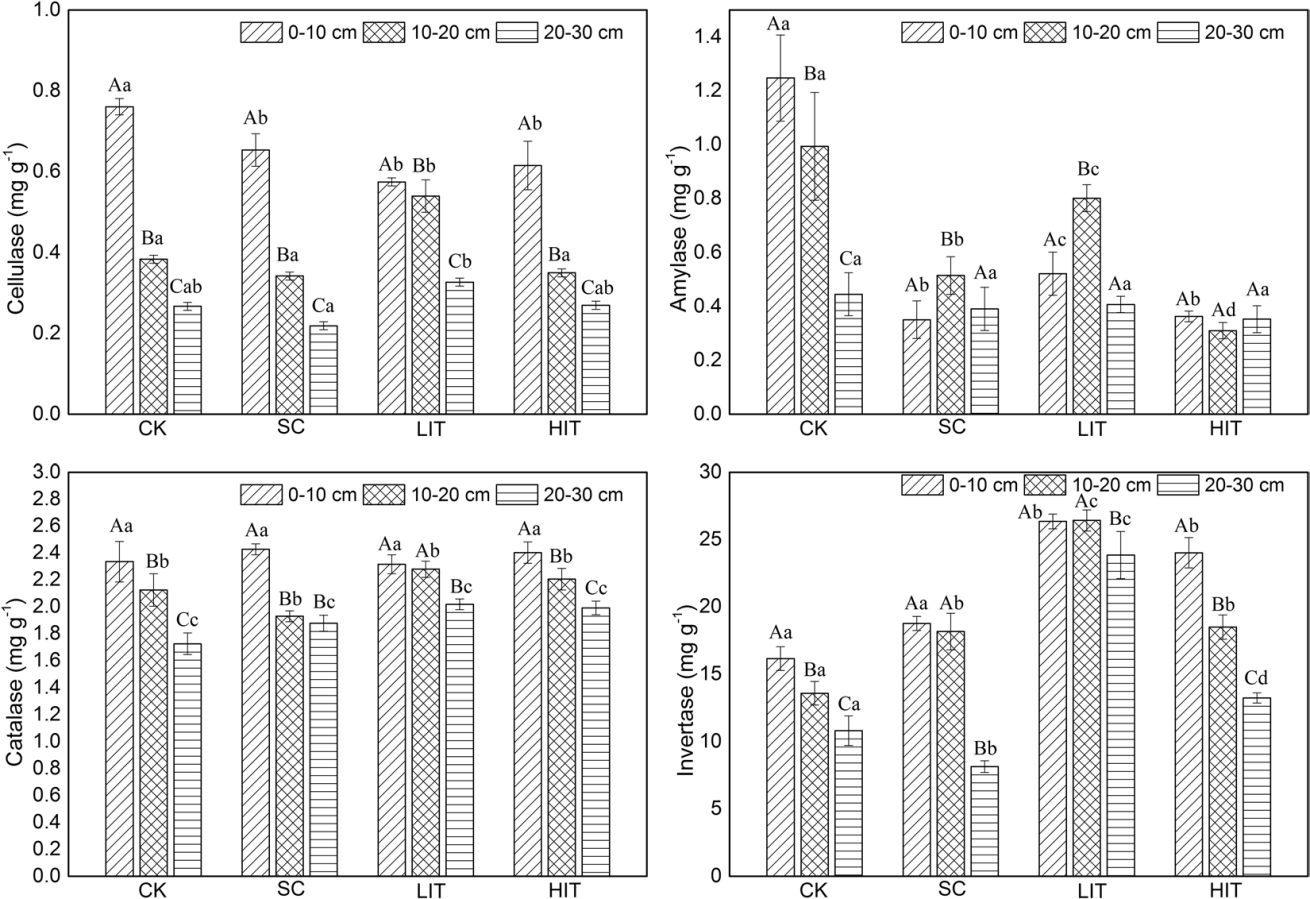
Soil enzymes in the four forest management treatments. The three columns in each treatment represent the quantities of four soil enzymes at different soil depths. Significant differences among different soil layers subjected to the same treatments are identified with A, B, and C (*p* < 0.05). Significant differences among different treatments of the same soil layer are identified with a, b, c, and d (*p* < 0.05), based on the analysis of variance. Values are means ± standard error (n = 3).

Soil enzyme activity decreased with increasing soil depth from the overall, which was consistent with the trend of vertical change in LOC content (Fig. 2). Soil cellulase enzyme activities in the 0–10 cm soil layer were higher than those in the other two soil layers (*p* < 0.05), and the activities of cellulase and invertase in the 10–20 cm soil layer were significantly higher than those in the 20–30 cm layer (*p* < 0.05).

Comparison with CK revealed that, the LIT and HIT treatment resulted in lower levels of cellulase, amylase, and catalase activity, whereas invertase activity increased. Cellulase activity in the 0–10 soil layer in the CK plots was significantly higher than that in the SC and thinning plots (*p* < 0.05). Amylase activity in the 0–10 and 10–20 cm soil layers in the CK plots were significantly higher than those in the LIT and HIT plots (*p* < 0.05). The differences of soil invertase activity between the LIT, HIT, and CK plot the in 10–20 cm and 20–30 cm layers were significant (*p* < 0.05).

### Relationships between soil LOC fractions and soil enzyme activities

SOC content was significantly positively correlated with the content of DOC, MBC, and EOC (r = 0.898, *p* < 0.01; r = 0.704, *p* < 0.01; r = 0.466, *p* < 0.01, respectively), as well as with the activities of cellulase, amylase, catalase, and invertase (r = 0.930, *p* < 0.01; r = 0.311, *p* < 0.05; r = 0.725, *p* < 0.01; r = 0.570, *p* < 0.05, respectively) (Table 2). The content of DOC, MBC, and EOC were significantly positively correlated with cellulase, catalase, and invertase activity and TN content (*p* < 0.01), but negatively correlated with TK and available phosphorous (AP) content, although this negative correlation was not significant). There was a significant positive correlation between DOC content and AK, NH_4_^+^–N, and NO_3_^−^–N content (r = 0.465, *p* < 0.01; r = 0.604, *p* < 0.01; r = 0.495, *p* < 0.01, respectively) and a negative correlation with pH (r = −0.308, *p* < 0.05). MBC content showed a significant positive correlation with AK and NH_4_^+^–N content (r = 0.337, *p* < 0.05 and r = 0.413, *p* < 0.01, respectively). There was significant correlation among DOC, MBC, and EOC, indicating that the components of LOC were closely related to each other. The activities of the four enzymes in this study were significantly correlated with the content of TN, NO_3_^−^–N, NH_4_^+^–N, and TP in the soil.

**Table 2:** Correlation of soil LOC content or enzyme activities with soil characteristics (n = 3). Significant correlations are indicated by *p < 0.05 or **p < 0.01 based on Pearson’s correlation analysis.

	SOC	DOC	MBC	ROC	Cellulase	Amylase	Catalase	Invertase	pH	TN	TP	TK	AP	AK	NH_4_^+^–N
DOC	0.898**														
MBC	0.704**	0.727**													
ROC	0.466**	0.460**	0.381**					^+^							
Cellulase	0.930**	0.866**	0.573**	0.434**											
Amylase	0.311*	0.088	−0.073	0.022	0.380**										
Catalase	0.725**	0.687**	0.619**	0.406**	0.710**	0.131									
Invertase	0.570*	0.541**	0.518**	0.384**	0.491**	0.016	0.558**								
pH	−0.359*	−0.308*	−0.019	−0.140	−0.464**	−0.421**	−0.366*	0.164							
TN	0.850**	0.861**	0.611**	0.395**	0.884**	0.311*	0.665**	0. 524*	−0.597**						
TP	0.679**	0.679**	0.423**	0.327*	0.721*	0.334*	0.423**	0.44*	−0.430**	0.862**					
TK	−0.130	−0.156	−0.026	−0.088	−0.227	−0.105	−0.328*	−0.335*	0.572**	−0.179	0.171				
AP	−0.201	−0.211	−0.035	−0.176	−0.254	−0.200	−0.157	0.199	0.520**	−0.329*	−0.208	0.268			
AK	0.635**	0.465**	0.337*	0.249	0.635**	0.588*	0.274	0.165	−0.287	0.599**	0.609**	0.131	−0.202		
NH_4_^+^–N	0.575**	0.604**	0.413**	0.032	0.628**	0.508*	0.531**	0.401*	−0.741**	0.785**	0.547**	−0.491**	−0.294*	0.313*	
NO_3_^−^–N	0.599**	0.495**	0.244	0.040	0.679**	0.597**	0.416**	−0.540*	−0.738**	0.793**	0.728**	−0.174	−0.293*	0.715**	0.771**

## Discussion

The content of soil TN, TP, AK, NH_4_^+^–N and NO_3_^−^–N decreased with increasing soil depth, and pH exhibited the opposite trend, which is consistent with previous findings^38–40^. According to Zhang *et al*.^39^, SOC and TN of a chestnut forest decreased after shrub cutting. In this study, contents of TN, AK, and NO_3_^−^–N in the 0–10, 10–20, and 20–30 cm soil layers were reduced in the LIT and HIT treatment. This might be due to a reduction in nutrient elements such as N, P, and calcium being returned to the soil through the litter, during to the thinning proces^41^. Moreover, we did not find any significant effect of treatment on soil pH and TP of all the soil layers, which were consistent with the findings of many studies^42–44^. This might be due to the positive and negative effects of the treatments on soil nutrients^42^. It is also possible that the effects of treatments on these properties will be manifested in the long term but not observed in the short period of this study^45^.

In this study, SOC content of soil (0–30 cm) in the CK, SC, LIT, and HIT treatment were 53.01, 53.84, 53.31, and 48.70 g kg^−1^, respectively, suggesting that HIT treatment reduced the SOC content remarkably, which was partially consistent with the hypothesis. Similar results were found by Chen *et al*.^12^ and Achat *et al*.^46^. The decrease in SOC content was mainly caused by the fact that, after the thinning, substrate inputs to the soil were reduced^3^. The microclimate induced an increase in the rate of SOC decomposition following decreased canopy closure and reduced SOC content^47^. In addition, the partial removal of tree canopies caused acceleration SOC leaching losses^12^. Moreover, we found that the response of SOC to thinning and understory vegetation removal was significantly different among three different soil layers because of the SOC in deeper soil layer with a longer residence time is less sensitive to disturbances^48^. These results may be due to the different compositions of plant species in different treatment plots wherein their roots reach different soil depths to control C sequestration and decomposition^49^.

SOC, DOC, MBC, and EOC content decreased with increasing soil depth, which is consistent with previous findings^8,50–52^. This may be related to the spatial distribution of root residual input and decomposition^52,53^. Although little is known about the composition of soil active C, the method has shown that it is more sensitive to soil management strategies than SOC is, and more closely related to soil biological properties^7,54–56^. According to Chatterjee *et al*.^57^ and Diochon *et al*.^58^, forest thinning causes in the return of a large amount of residual organic matter to the surface, accompanied by changes in light conditions, which can lead to significant changes in LOC mineralisation. In our work, the contents of soil EOC, MBC, and DOC in thinning treatments were higher than those in the CK plots was consistent with our hypothesis; particularly, the HIT treatment significantly increased these contents in the 0–10 cm layer and the effect was more marked in the upper than in the lower soil layers. The result was supported by the findings from a study on coniferous forests in Wyoming, USA^57^ and those of Chen *et al*.^12^. However, high-intensity thinning promoted non-tree vegetation owing to the sparse forest canopy. Subsequently, their fine roots would increase the LOC input by root exudates. In addition, high-intensity thinning accelerated the decomposition of litter and residue, and the accumulation of LOC fractions, consequently the potential SOC mineralisation rate increased^12,59^.

Soil enzymes participate in almost every transformation process of litter decomposition and play a central role in maintaining forest soil fertility by releasing plant available mineral nutrients from complex organic resources^60,61^. Soil invertase, cellulase and catalase activities decreased with increasing soil depth, in accordance with the results of Xiao *et al*.^52^ and Chen *et al*.^12^. A high content of organic matter in surface soil is beneficial to the growth of microorganisms with active metabolic processes, which in turn leads to the accumulation of soil enzymes in the surface layer. In this study, the overall cellulase and amylase activities decreased after thinning, which is supported by Chen *et al*.^12^. Similar results were observed in the New Jersey Pine Barrens, where cellulase and phenol oxidase activities significantly decreased after one year of thinning^62^. The results were partially consistent with hypothesis of this study. These variations in enzyme activities can be due to reductions in root activity and changes in microbial composition. Furthermore, the extent of utilisation of the C and N sources for soil enzymes differs, with varying effects on soil enzyme activity under different management strategies. Li *et al*.^63^ reported that invertase can break down some carbohydrate polymers to release the nutrients from organic compounds through its role in the first phases of degradation of organic compounds. During this phase, molecular size is reduced and smaller organic structures are produced, which facilitates microbial enzyme activities. We found that the thinning treatments had greater invertase activity than that in the CK treatment. A plausible explanation is that invertase activity is positively correlated with soil pH, TN, and TP, but it is negatively correlated with TK and NO_3_^−^–N.

We found that the SOC content was significantly correlated with the four enzymes activities and soil LOC components, which was observed in earlier studies^63,64^. The result suggested that the soil LOC components and these enzymes activities were sensitive to the variations of SOC which was consistent with the hypothesis. Significant correlations were found among the LOC fractions and invertase, catalase, and cellulase activities in the soil, which is consistent with findings of Paz-Ferreior^65^. In addition, significant correlations were found between each component of SOC and the soil TN content in the soil, consistent with findings of Geng *et al*.^62^. These correlations might have arisen because the N content in the soil organic matter affects the rate of soil organic matter decomposition and consumption by microorganisms. Nitrogen-rich organic matter is easily and rapidly decomposed, transferred, and converted by microorganisms, thus increasing the SOC content in the soil. As soil enzymes directly participate in the utilisation of soil nutrients, they indirectly reflect the dynamic state of the conversion of soil nutrients. Taken together, a similar conclusion to that of Ma *et al*.^66^ can be drawn in that enhancing soil nutrient content is the key factor for increasing the accumulation of LOC fractions.

## Conclusions

This study demonstrates the distribution of chemical properties of soil, LOC fractions, and enzyme activities, and provides insight into their relationships in *Pinus massoniana* plantations under different forest management approaches. Our results showed that the content of soil LOC fractions, TN, TP, AK, NH_4_^+^–N, NO_3_^−^–N and enzyme activities decreased with increasing soil depth in all the treatments. High-intensity thinning reduced the SOC content remarkably. Soil EOC, DOC, and MBC contents were higher in thinning treatments than those in the control; especially high-intensity thinning treatment significantly increased those contents in 0–10 cm layer. Simultaneously, thinning resulted in a decrease in cellulase and amylase activities, but an increase in invertase activity. The variations of SOC, EOC, DOC, MBC, cellulose, amylase, and invertase were more marked in the top soil layer than in the deeper soil layers. The correlation analysis showed that the soil LOC components and enzymes activities were sensitive to the variations in SOC. Our results indicate that high-intensity thinning treatments in *Pinus massoniana* plantation decreased the SOC content and might lead to an increase of LOC components, in which the effect was more marked in the 0–10 cm soil layer than in the deeper layers.

## Materials and Methods

### Study site

**Figure 3:**
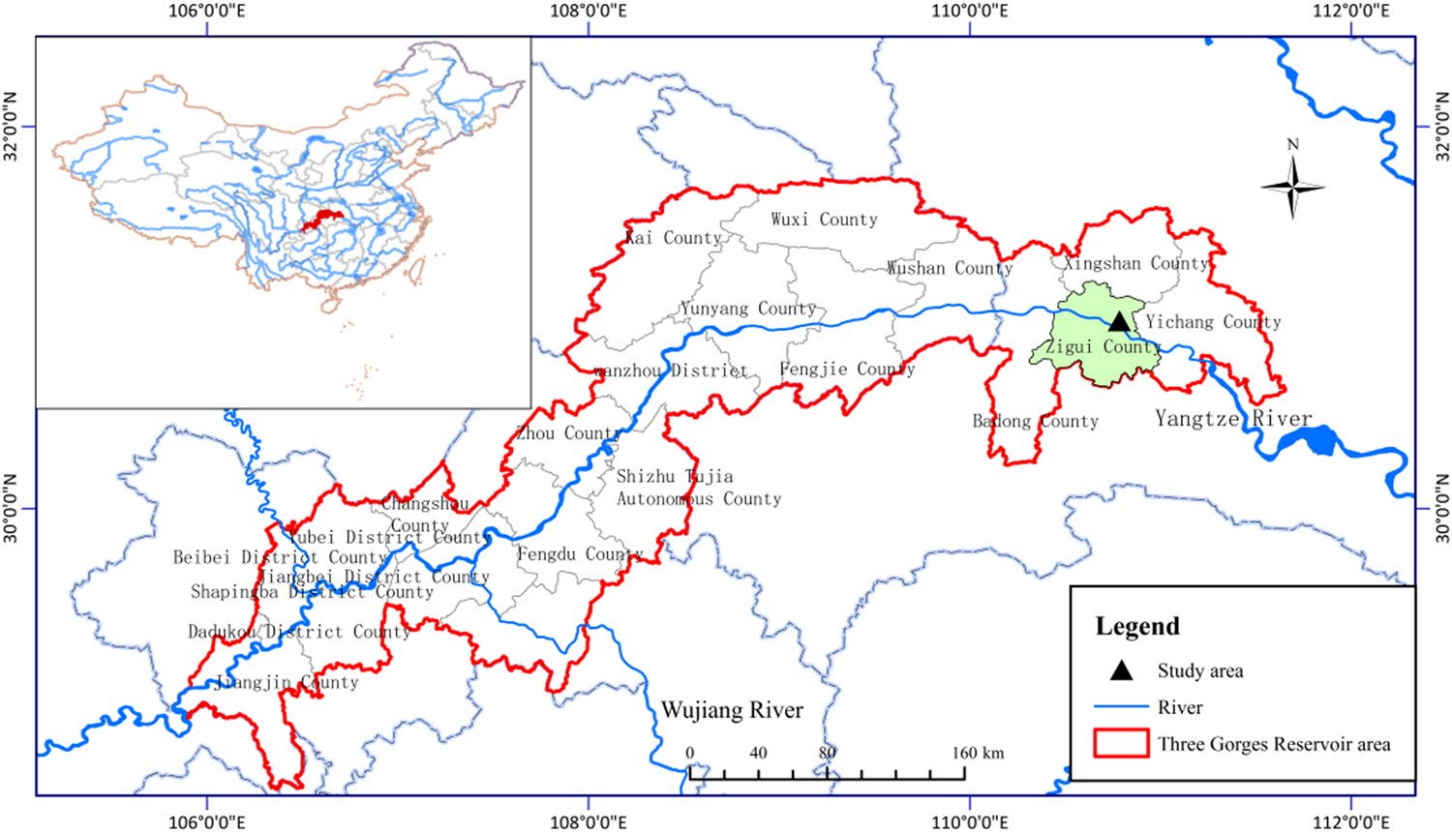
Location of the study area. The study area, located in the Jiulingtou Forest Farm (30°59′N, 110°47′E), is indicated by a triangle and Zigui City is marked in green. The blue line represents the Yangtze River flowing through the Three Gorges Reservoir area (the region surrounded by the red line). The maps were created using ArcGIS 10.2 software (ESRI 2014).

The experimental plots were located in the Jiulingtou Forest in Zigui County, Hubei Province, China, where *Pinus massoniana* were aerially seeded in the 1970s (Fig. 3). The soil type of the plots was haplic luvisol^67^, and the annual mean temperature was approximately 16.9 °C with annual rainfall varying between 1000 and 1250 mm, occurring primarily from April to September^68^. Twelve 20 m × 20 m plots within *Pinus massoniana* monoculture plantations, laid out in an orthogonal design and separated from each other by at least 2 m, were defined in September 2013. Each plot contained four treatments, randomly assigned per row. Treatments consisted of (1) intact forest (control, CK), (2) all shrubs harvested and residual materials removed (shrub cutting, SC), (3) low-intensity thinning (LIT) with 15% of the basal area of the trees removed, and (4) high-intensity thinning (HIT) with 70% of the basal area of the trees removed. The study area was relatively steep with a northwest-facing slope of 34°. The experiment was conducted in mid-October 2013 and included a control intact forest, and thinning and shrub cutting treatments using chain saws. Only the harvested trunks were removed and the residue produced by the harvest was not cleared from the plots. Except for plots subjected to the SC treatment, the understory shrub layer was dominated by *Lespedeza bicolor* Turcz., *Pyracantha fortuneana* (Maxim.) Li, and *Litsea pungens* Hemsl., with an average diameter at breast height of 5.00 cm and average height of 5.60 m. Herbs in the study area were mainly *Woodwardia japonica* L.f., *Carex tristachya* Thunb., *Aster ageratoides* Turcz., and *Parathelypteris nipponica* (Franchet and Savatier) Ching.

### Soil analysis

The twelve plots (4 treatments × 3 replications per treatment) were divided into 24 subplots (10 m × 20 m). Six randomly-placed replicate soil samples were collected from each subplot in June 2016 at depths of 0–10, 10–20, and 20–30 cm yielding 432 (24 subplots × 6 replicates per subplot × 3 soil layers) samples, which were collected using a soil sampler 30 cm in length. These samples were placed in plastic bags and stored in a portable cooler for transport to the laboratory. The soil samples were divided into two subsamples of equal volume. One was passed through a 2-mm sieve to remove impurities of soil and stored at 4 °C before testing. This subsample was used to determine NH_4_^+^–N, NO_3_^−^–N, DOC, MBC, EOC, and enzyme activities (cellulase, amylase, invertase, and catalase). The other subsample was air-dried and sieved before use for the analysis of SOC and other soil properties (TN, TP, TK, AK, AP, and pH).

### Sample analyses

#### Soil chemical analysis

SOC content was measured using dichromate oxidation^69^. Soil TN was determined using the Kjeldahl method^70^. NH_4_^+^–N and NO_3_^−^–N concentrations were determined using a flow injection analyser, TP, TK, AK, and AP were measured using inductively coupled plasma mass spectrometry (ICP-MS) analysis (IRIS Intrepid II XSP system; Thermo Electric Co., USA). Soil pH was determined from a soil water (1:5 w/v) suspension, prepared by shaking 30 min, using a conductivity meter.

Total DOC content was measured using dichromate oxidation titration^71^, and EOC content was analysed using 0.333 mol L^−1^ KMnO_4_ oxidation^72^. MBC content was measured using chloroform fumigation extraction^73^.

#### Soil enzyme activity analysis

Soil amylase activity was measured using 2 g of fresh soil incubated for 24 h at 37 °C according to Ebregt’s method^74^. Soil invertase activity was measured as at 30 °C and pH 4.65 in Na-acetate buffer according to Gianfreda’s method^75^. Soil cellulase activities were detected by an incubation according to Sharma’s method^76^, and soil catalase activity was determined at pH 7.0, following the monitoring of the decomposition of H_2_O_2_ at 240 nm with an extinction coefficient of 43.6 M^−1^cm^−1^ according to Roggenkamp and Sahm^77^.

### Data analyses

All data were analysed using SPSS 22.0 for Windows (SPSS Inc., Chicago, IL, USA). One-way analyses of variance (ANOVA) and comparisons among means were made using the least significant difference (LSD) test, with *p* < 0.05 regarded as significant. Pearson’s correlation coefficients of soil LOC fractions (EOC, DOC, and MBC) with enzyme activities (invertase, cellulase, catalase, and amylase) and other soil characteristics were estimated, with a significance level of *p* < 0.05.

## References

[1] Six J (2002). Measuring and understanding carbon storage in afforested soils by physical fractionation. Soil science society of America journal.

[2] Lal R (2004). Soil carbon sequestration to mitigate climate change. Geoderma.

[3] Jandl R (2007). How strongly can forest management influence soil carbon sequestration?. Geoderma.

[4] Mckinley DC (2011). A synthesis of current knowledge on forests and carbon storage in the united states. Ecological Applications.

[5] Moreno-Fernández D (2015). Temporal carbon dynamics over the rotation period of two alternative management systems in mediterranean mountain scots pine forests. Forest Ecology & Management.

[6] Haynes RJ (2005). Labile organic matter fractions as central components of the quality of agricultural soils: an overview. Advances in Agronomy.

[7] Cai A, Feng W, Zhang W, Xu M (2016). Climate, soil texture, and soil types affect the contributions of fine-fraction-stabilized carbon to total soil organic carbon in different land uses across China. Journal of Environmental Management.

[8] Li YF (2013). Long-term intensive management effects on soil organic carbon pools and chemical composition in Moso bamboo (Phyllostachys pubescens) forests in subtropical China. Forest Ecology and Management.

[9] Rovira P, Vallejo VR (2007). Labile, recalcitrant, and inert organic matter in Mediterranean forest soils. Soil Biology & Biochemistry.

[10] Ghani A, Dexter M, Perrott KW (2003). Hot-water extractable carbon in soils: a sensitive measurement for determining impacts of fertilisation, grazing and cultivation. Soil Biology & Biochemistry.

[11] Zou XM, Ruan HH, Fu Y, Yang XD, Sha LQ (2005). Estimating soil labile organic carbon and potential turnover rates using a sequential fumigation–incubation procedure. Soil Biology & Biochemistry.

[12] Chen X (2016). Soil labile organic carbon and carbon-cycle enzyme activities under different thinning intensities in Chinese fir plantations. Applied Soil Ecology.

[13] Mosca E, Montecchio L, Scattolin L, Garbaye J (2007). Enzymatic activities of three ectomycorrhizal types of *Quercus robur L. in relation to tree d*ecline and thinning. Soil Biology & Biochemistry.

[14] Boerner RE, Waldrop TA, Shelburne VB (2006). Wildfire mitigation strategies affect soil enzyme activity and soil or Canadian. Journal of Forest Research.

[15] Verschuyl J, Riffell S, Miller D, Wigley TB (2011). Biodiversity response to intensive biomass production from forest thinning in north american forests – a meta-analysis. Forest Ecology & Management.

[16] Fu X (2015). Understory vegetation leads to changes in soil acidity and in microbial communities 27 years after reforestation. Science of the Total Environment.

[17] Blanco JA, Imbert JB, Castillo FJ (2006). Influence of site characteristics and thinning intensity on litterfall production in two *Pinus sylvestris L. forests in the western Pyren*ees. Forest Ecology & Management..

[18] Roig S, Río MD, Cañellas I, Montero G (2005). Litter fall in Mediterranean Pinus pinaster Ait. stands under different thinning regimes. Forest Ecology & Management.

[19] Slodicak M, Novak J, Skovsgaard JP (2005). Wood production, litter fall and humus accumulation in a Czech thinning experiment in Norway spruce (*Picea abies (L.) Karst.)*. Forest Ecology & Management.

[20] Prescott CE (1997). Effects of clearcutting and alternative silvicultural systems on rates of decomposition and nitrogen mineralization in a coastal montane coniferous forest. Forest Ecology & Management.

[21] Yin X, Perry JA, Dixon RK (1989). Influence of canopy removal on oak forest floor decomposition. Canadian Journal of Forest Research.

[22] Bravo F, Bravo-Oviedo A, Diaz-Balteiro L (2008). Carbon sequestration in Spanish Mediterranean forests under two management alternatives: a modeling approach. European. Journal of Forest Research.

[23] Lindner M, Karjalainen T (2007). Carbon inventory methods and carbon mitigation potentials of forests in Europe: a short review of recent progress. European Journal of Forest Research.

[24] Wu J (2011). Understory plants can make substantial contributions to soil respiration: Evidence from two subtropical plantations. Soil Biology & Biochemistry.

[25] Skovsgaard JP, Stupak I, Vesterdal L (2006). Distribution of biomass and carbon in even-aged stands of Norway spruce (*Picea abies* (L.) Karst.): a case study on spacing and thinning effects in northern Denmark Scand. J. For. Res..

[26] Jiménez, E., Vega, J. A., Fernández, C. & Fonturbel, T. Is pre-commercial thinning compatible with carbon sequestration? A case study in a maritime pine stand in northwestern Spain. *Forestry*, 1–9 (2011)

[27] Dwyer JM, Fensham R, Buckley YM (2010). Restoration thinning accelerates structural development and carbon sequestration in an endangered Australian ecosystem. Applied Soil Ecology.

[28] Horner GJ (2010). Forest structure, habitat and carbon benefits from thinning floodplain forests: managing early stand density makes a difference. For. Ecol. Manage.

[29] De LHJ, Moya D, López-Serrano FR, Rubio E (2013). Carbon sequestration of naturally regenerated Aleppo pine stands in response to early thinning. New Forest.

[30] Turner MG, Whitby TG, Tinker DB, Romme WH (2016). Twenty-four year after the Yellowstone Fires: are postfire lodgepole pine stand converging in structure and function?. Ecology.

[31] Guo LJ, Zhang ZS, Wang DD, Li CF, Cao CG (2015). Effects of short-term conservation management practices on soil organic carbon fractions and microbial community composition under a rice-wheat rotation system. Biology & Fertility of Soils.

[32] Guang QL (2016). Carbon stock of Larch plantations and its comparison with an old-growth forest in northeast China. Chinese Geographical Science.

[33] Kris V (2016). Contributions of a global network of tree diversity experiments to sustainable forest plantations. Ambio.

[34] Wang H (2010). Soil organic carbon stock and chemical composition in four plantations of indigenous tree species in subtropical China. Ecological Research.

[35] Wang RL (2012). Fine root production and turnover in Pinus massoniana plantation in Three Gorges Reservoir area of China. Chinese Journal of Applied Ecology.

[36] Wang XJ (2014). Estimation of forest productivity and carbon storage in Three Gorges Reservoir. Ecological Science.

[37] Wang PC (2009). Organic carbon density and storage of forest ecosystems in three gorges reservoir area. Acta Ecologica Sinica.

[38] Bobuľská L, Fazekašová D, Angelovičová L (2015). Vertical Profiles of Soil Properties and Microbial Activities in Peatbog Soils in Slovakia. Environmental Processes.

[39] Zhang J (2014). Understory vegetation management affected greenhouse gas emissions and labile organic carbon pools in an intensively managed chinese chestnut plantation. Plant & Soil.

[40] Zhang S, Huffman T, Zhang X, Liu W, Liu Z (2014). Spatial distribution of soil nutrient at depth in black soil of Northeast China: a case study of soil available phosphorus and total phosphorus. Journal of Soils and Sediments.

[41] Blanco JA, Imbert JB, Castillo FJ (2011). Thinning affects Pinus sylvestris needle decomposition rates and chemistry differently depending on site conditions. Biogeochemistry.

[42] Xiong Y, Fu S (2008). Impacts of litter and understory removal on soil properties in a subtropical acacia mangium plantation in china. Plant & Soil.

[43] Bravooviedo A, Ruiz-Peinado R, Modrego P, Alonso R, Montero G (2015). Forest thinning impact on carbon stock and soil condition in southern european populations of *P. sylvestris l*. Forest Ecology & Management.

[44] Wang F, Zou B, Li H, Li Z (2014). The effect of understory removal on microclimate and soil properties in two subtropical lumber plantations. Journal of Forest Research.

[45] Heenan DP, Chan KY, Knight PG (2004). Long-term impact of rotation, tillage and stubble management on the loss of soil organic carbon and nitrogen from a chromic luvisol. Soil & Tillage Research.

[46] Achat DL, Fortin M, Landmann G, Ringeval B, Augusto L (2015). Forest soil carbon is threatened by intensive biomass harvesting. Scientific Reports.

[47] Piene H, Cleve KV (1978). Weight loss of litter and cellulose bags in a thinned white spruce forest in interior Alaska. Canadian Journal of Forest Research.

[48] Fontaine S (2007). Stability of organic carbon in deep soil layers controlled by fresh carbon supply. Nature.

[49] Clemmensen KE (2013). Roots and associated fungi drive long-term carbon sequestration in boreal forest. Science.

[50] Cheng X (2008). Assessing the Effects of Short-term Spartina alterniflora Invasion on Labile and Recalcitrant C and N pools by Means of Soil Fractionation and Stable C and N Isotopes. Geoderma.

[51] Wang, G., Ma, A. & Xia, Y. Distribution of soil active organic carbon under different management. *Patterns of Poplar Plantation*, 1771–1776 (2015).

[52] Xiao Y, Huang Z, Lu X (2015). Changes of soil labile organic carbon fractions and their relation to soil microbial characteristics in four typical wetlands of Sanjiang Plain, Northeast China. Ecological Engineering.

[53] Ecosystem Carbon and Nitrogen Stocks in the Yangtze Estuary, *China Ecosystems* **10**, 1351–1361 (2007)

[54] Dodla SK, Wang JJ, Delaune RD (2012). Characterization of labile organic carbon in coastal wetland soils of the Mississippi River deltaic plain: relationships to carbon functionalities. The Science of the total environment.

[55] Dodla SK, Wang JJ, Delaune RD, Breitenbeck G (2009). Carbon gas production under different electron acceptors in a freshwater marsh soil. Chemosphere.

[56] Zhang J, Song C, Yang W (2006). Land use effects on the distribution of labile organic carbon fractions through soil profiles. Soil Science Society of America Journal.

[57] Chatterjee A, Vance GF, Pendall E, Stahl PD (2008). Timber harvesting alters soil carbon mineralization and microbial community structure in coniferous forests. Soil Biology & Biochemistry.

[58] Diochon A, Kellman L, Beltrami H (2009). Looking deeper: An investigation of soil carbon losses following harvesting from a managed northeastern red spruce (Picea rubens Sarg.) forest chronosequence. Forest Ecology & Management.

[59] Li CF (2012). Short-term effects of conservation management practices on soil labile organic carbon fractions under a rape–rice rotation in central china. Soil & Tillage Research.

[60] Baldrian, P. & Štursová, M. Enzymes in Forest Soils. *Springer Berlin Heidelberg* (2011)

[61] Adamczyk B, Adamczyk S, Kukkola M, Tamminen P, Smolander A (2015). Logging residue harvest may decrease enzymatic activity of boreal forest soils. Soil Biology & Biochemistry.

[62] Geng Y, Dighton J, Gray D (2012). The effects of thinning and soil disturbance on enzyme activities under pitch pine soil in New Jersey Pinelands. Applied Soil Ecology.

[63] Li S (2016). Dynamics of soil labile organic carbon fractions and C-cycle enzyme activities under straw mulch in Chengdu Plain. Soil Tillage Research.

[64] Xu Z (2015). The variations in soil microbial communities, enzyme activities and their relationships with soil organic matter decomposition along the northern slope of Changbai Mountain. Applied Soil Ecology.

[65] Paz-Ferreiro J, Trasar-Cepeda C, Leirós MDC, Seoane S, Gil-Sotres F (2011). Intra-annual variation in biochemical properties and the biochemical equilibrium of different grassland soils under contrasting management and climate. Biology & Fertility of Soils.

[66] Ma S, Li Z, Wang B, Liu R, Wang G (2012). Changes in soil active organic carbon under different management types of bamboo stands. Acta Ecologica Sinica.

[67] Gong ZT (2003). Chinese Soil Taxonomy.

[68] Xiao WF (2014). Rates of litter decomposition and soil respiration in relation to soil temperature and water in different-aged *Pinus massoniana* forests in the Three Gorges Reservoir Area, China. Plos One.

[69] Nelson D. W. *et al*. Total carbon, organic carbon, and organic matter Methods of Soil Analysis Part—chemical Methods, 961–1010 (1982).

[70] Zhu T (2014). Tea plantation destroys soil retention of NO_3_ − and increases N_2_*O emissions in subtropical China*. Soil Biology & Biochemistry.

[71] Ciavatta C, Govi M, Antisari LV, Sequi P (1991). Determination of organic carbon in aqueous extracts of soils and fertilizers Communications in Soil. Science & Plant Analysis.

[72] Blair GJ, Lefroy R, Lisle L (1995). Soil carbon fractions based on their degree of oxidation, and the development of a carbon management index for agricultural systems. Australian. Journal of Agricultural Research.

[73] Vance ED, Brooks PC, Jenkinson DS (1987). An Extraction Method for Measuring Soil Microbial Biomass. Soil Biology & Biochemistry.

[74] Ebregt A, Boldewijn JMAM (1977). Influence of heavy metals in spruce forset soil on amylase activity, CO_2_ evolution from starch and soil respiration. Plant & Soil.

[75] Gianfreda L, Sannino F, Violante A (1995). Pesticide effects on the activity of free, immobilized and soil invertase. Soil Biology & Biochemistry.

[76] Sharma N, Bhalla TC, Bhatt AK (1991). Partial purification and characterization of extracellular cellulase from a strain of Trichoderma viride isolated from forest soil. Folia Microbiologica.

[77] Roggenkamp R, Sahm H, Wagner F (1974). Microbial assimilation of methanol induction and function of catalase in Candida boidinii. FEBS Lett.

