# Author Correction: Labile organic carbon pools and enzyme activities of *Pinus massoniana* plantation soil as affected by understory vegetation removal and thinning

**DOI:** 10.1038/s41598-018-29781-0

**Published:** 2018-07-25

**Authors:** Yafei Shen, Ruimei Cheng, Wenfa Xiao, Shao Yang, Yan Guo, Na Wang, Lixiong Zeng, Lei Lei, Xiaorong Wang

**Affiliations:** 1 Key Laboratory of Forest Ecology and Environment, State Forestry Administration, Research Institute of Forest Ecology, Environment and Protection, Chinese Academy of Forestry, Beijing, 100091 China; 2 Co-innovation Center for Sustainable Forestry in Southern China, Nanjing Forestry University, Nanjing, 210037 China

Correction to: *Scientific Reports* 10.1038/s41598-017-18812-x, published online 12 January 2018

The Article contains errors in Table 1. The correct Table 1 appears below.

**Table 1:** Soil chemical properties at three soil depths in the four forest management treatments (mean value ± standard error; n = 3).

Treat-ments	Soil depth (cm)	Soil pH	TN (g kg^−1^)	TP (g kg^−1^)	TK (g kg^−1^)	AP (mg kg^−1^)	AK (mg kg^−1^)	NO_3_^−^–N (mg kg^−1^)	NH_4_^+^–N (mg kg^−1^)
CK	0–10	5.85 ± 0.02 Aa	1.65 ± 0.01 Aa	0.21 ± 0.01 Aa	17.05 ± 0.12 Aa	0.84 ± 0.10 a	184.68 ± 2.19 Aa	17.79 ± 0.89 Aa	43.00 ± 3.51 Aa
	10–20	5.92 ± 0.06 Ba	1.15 ± 0.01 Ba	0.18 ± 0.01 Ba	16.76 ± 0.13 Ba	0.99 ± 0.17 a	145.83 ± 2.80 Ba	11.26 ± 0.99 Ba	37.34 ± 3.48 Ba
	20–30	6.07 ± 0.05 Ca	0.97 ± 0.01 Ca	0.17 ± 0.01 Ba	17.13 ± 0.10 Aa	0.98 ± 0.17 a	136.58 ± 0.47 Ca	5.40 ± 0.0.37 Ca	24.66 ± 0.56 Ca
SC	0–10	6.02 ± 0.05 Aa	1.57 ± 0.02 Ab	0.20 ± 0.01 Aa	18.21 ± 0.15 Ab	1.23 ± 0.17Aa	146.45 ± 2.04 Ab	11.65 ± 0.22 Ab	45.66 ± 0.69 Aa
	10–20	6.16 ± 0.03 Ba	1.16 ± 0.01 Ba	0.19 ± 0.01 Ba	18.08 ± 0.25 Bb	2.35 ± 0.29 Bb	126.18 ± 1.28 Bb	4.48 ± 0.14 Bb	18.96 ± 0.23 Bb
	20–30	6.33 ± 0.03 Ca	0.90 ± 0.01 Cb	0.18 ± 0.01 Ca	19.52 ± 0.19 Cab	1.57 ± 0.4 ABab	110.43 ± 2.47 Cb	2.75 ± 0.34 Cb	12.76 ± 1.28 Cb
LIT	0–10	6.17 ± 0.02 Aa	1.37 ± 0.04 Ac	0.19 ± 0.01 Aa	16.54 ± 0.21 Ac	2.11 ± 0.39 b	140.60 ± 3.29 Acb	9.73 ± 0.42 A c	50.69 ± 2.91 Ab
	10–20	6.25 ± 0.03 Ba	1.04 ± 0.02 Bb	0.17 ± 0.01 Ba	17.14 ± 0.08 Aa	1.46 ± 0.92 ac	127.40 ± 2.50 Bb	2.57 ± 0.10 Bc	14.79 ± 0.47 Bc
	20–30	6.48 ± 0.02 Ca	0.85 ± 0.0 1 Cc	0.17 ± 0.01 Ba	17.70 ± 0.24 Aab	2.51 ± 0.61 b	126.25 ± 3.29 Aa	2.45 ± 0.13 Bb	10.99 ± 0.28 Cb
HIT	0–10	5.97 ± 0.05 Aa	1.49 ± 0.01 Ad	0.19 ± 0.01 Aa	16.43 ± 0.16 Acd	0.82 ± 0.70 Aa	130.78 ± 0.52 Ac	5.35 ± 0.19 Ad	35.79 ± 3.29 Ac
	10–20	6.07 ± 0.03 Ba	1.09 ± 0.02 Bc	0.17 ± 0.01 Ba	15.83 ± 0.34 Bc	1.09 ± 0.10 ABad	82.90 ± 1.60 Bc	4.90 ± 0.58 ABb	34.51 ± 2.41 Aa
	20–30	6.20 ± 0.04 Ca	0.96 ± 0.01 Ca	0.18 ± 0.01 Ca	16.58 ± 0.05 Cb	1.91 ± 0.90 Bab	71.68 ± 1.57 Cc	3.50 ± 1.56 Bb	29.25 ± 1.50 Bc

Significant differences among different soil layers subjected to the same treatments are identified with A, B, and C (*p* < 0.05). Significant differences among different treatments of the same soil layer are identified with a, b, c, and d (p < 0.05), based on the analysis of variance.

This Article contains errors in the labelling and scaling of the charts in Figure 1. The correct Figure 1 appears below:

**Figure 1:**
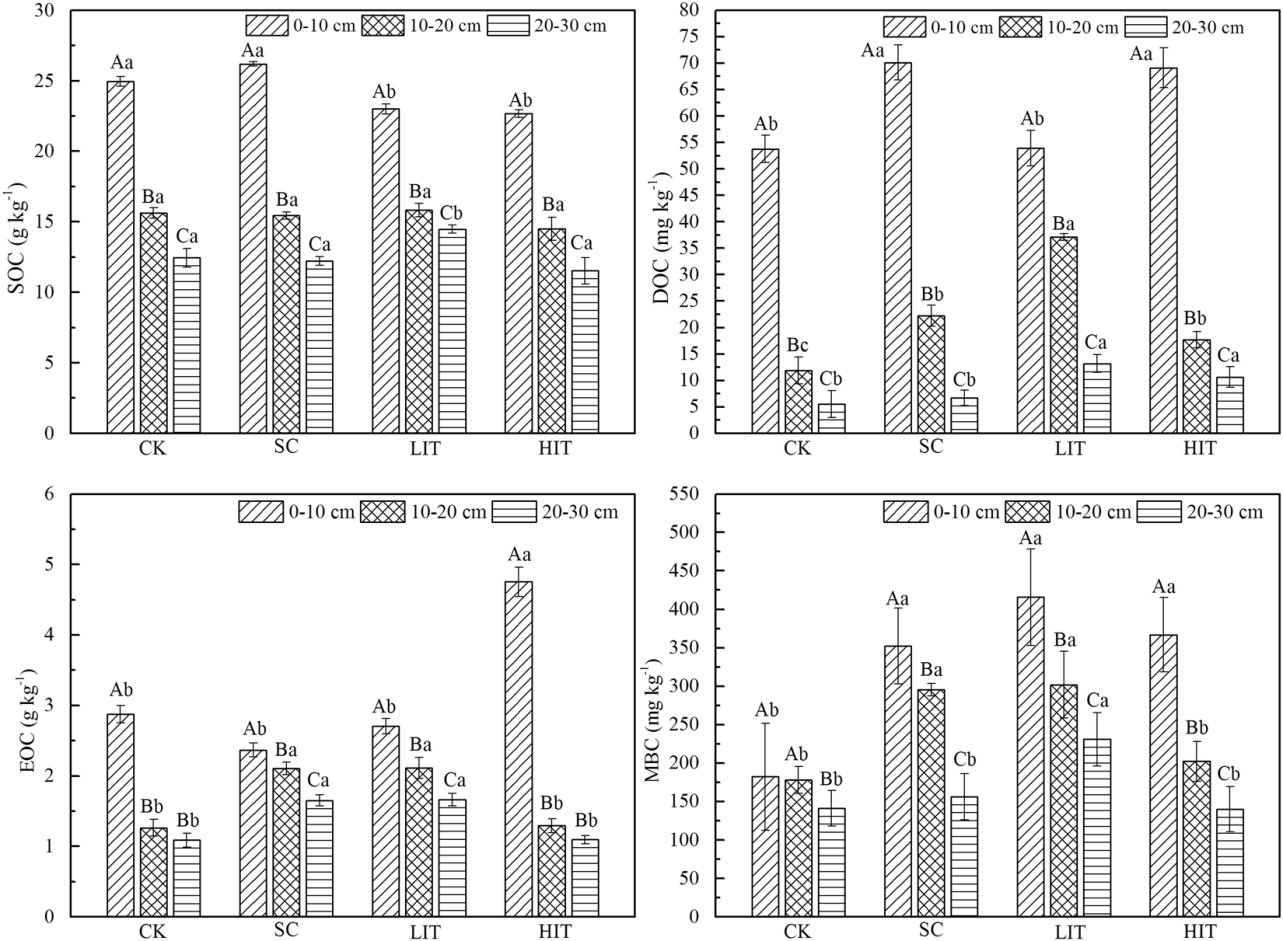
Soil LOC fractions in the four forest management treatments. The three columns in each treatment represent the quantities in soil LOC content at different soil depths. Significant differences among different soil layers subjected to the same treatments are identified with A, B, and C (p < 0.05). Significant differences among different treatments of the same soil layer are identified with a, b, c, and d (p < 0.05), based on the analysis of variance. Values are means ± standard error (n = 3).

In addition, the Article also contains errors in the units and labelling for the charts in Figure 2. The correct Figure 2 appears below:

**Figure 2:**
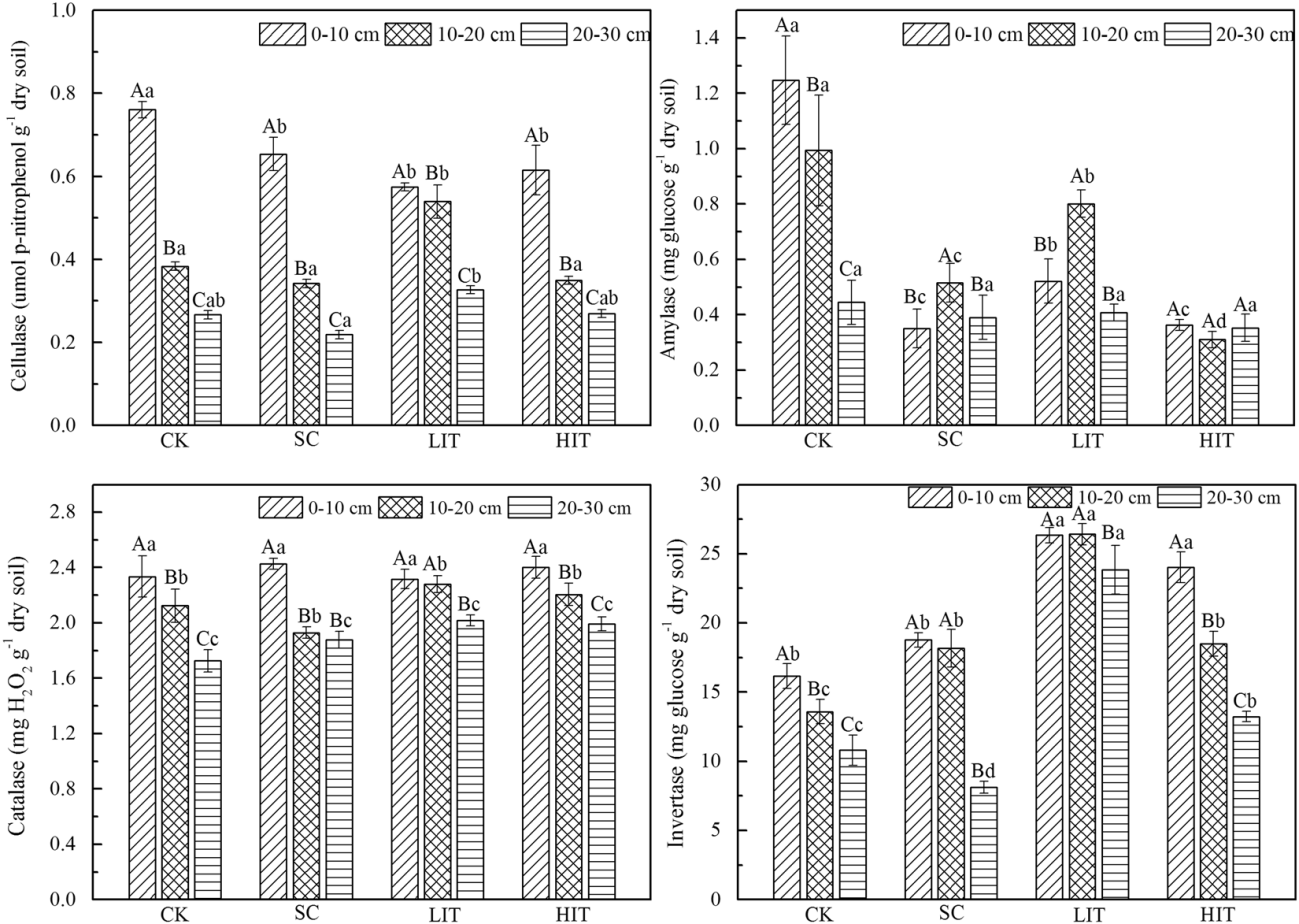
Soil enzymes in the four forest management treatments. The three columns in each treatment represent the quantities of four soil enzymes at different soil depths. Significant differences among different soil layers subjected to the same treatments are identified with A, B, and C (p < 0.05). Significant differences among different treatments of the same soil layer are identified with a, b, c, and d (p < 0.05), based on the analysis of variance. Values are means ± standard error (n = 3).

The Article also contains errors in Table 2 where EOC was incorrectly given as ROC. In addition, Table 2 contains a typographical error in the fourth row of the ‘Invertase’ column where ‘+’ is erroneously present.

This Article contains errors in the Materials and Methods section under subheading ‘*Soil analysis’*.

“This subsample was used to determine NH_4_^+^–N, NO_3_^−^–N, DOC, MBC, EOC, and enzyme activities (cellulase, amylase, invertase, and catalase). The other subsample was air-dried and sieved before use for the analysis of SOC and other soil properties (TN, TP, TK, AK, AP, and pH).”

should read:

“This subsample was used to determine DOC, MBC, EOC, and enzyme activities (cellulase, amylase, invertase, and catalase). The other subsample was air-dried and sieved before use for the analysis of SOC and other soil properties (TN, NH_4_^+^–N, NO_3_^−^–N, TP, TK, AK, AP, and pH).”

Also under the subheading ‘*Sample analyses’*.

“Soil pH was determined from a soil water (1:5 w/v) suspension, prepared by shaking 30 min, using a conductivity meter.”

should read:

“Soil pH was determined from a soil water (1:2.5) suspension, prepared by shaking 30 min, using a conductivity meter.”

Finally, the authors neglected to cite a previously-published related paper. This is listed below as reference^1^.

As a result, in the Materials and Methods section under subheading ‘Soil enzyme activity analysis’

“Soil amylase activity was measured using 2 g of fresh soil incubated for 24 h at 37 °C according to Ebregt’s method^74^. Soil invertase activity was measured as at 30 °C and pH 4.65 in Na-acetate buffer according to Gianfreda’s method^75^. Soil cellulase activities were detected by an incubation according to Sharma’s method^76^, and soil catalase activity was determined at pH 7.0, following the monitoring of the decomposition of H_2_O_2_ at 240 nm with an extinction coefficient of 43.6 M^−1^ cm^−1^ according to Roggenkamp and Sahm^77^.”

should read:

“Soil amylase activity was measured using 2 g of fresh soil incubated for 24 h at 37 °C according to methods of Ebregt^74^ and Guan^1^. Soil invertase activity was measured according to Guan’s method^1^ and Gianfreda’s method^75^. The amylase and invertase activities were expreseed as mg glucose g^−1^ soil 24 h^−1^. Soil cellulase activities were detected by an incubation according to Sharma’s method^76^ and Guan’s method^1^, and the activity was expreseed as μmol p-nitrophenol g^−1^ soil h^−1^. Soil catalase activity was determined at pH 7.0, following the monitoring of the decomposition of H_2_O_2_ at 240 nm with an extinction coefficient of 43.6 M^−1^ cm^−1^ according to Roggenkamp^77^ and Guan^1^, and the activity was expreseed as mg H_2_O_2_ g^−1^ soil 20 min^−1^.”
